# Brucellosis in Patients with Crimean-Congo Hemorrhagic Fever

**Published:** 2017-12-30

**Authors:** Fazilet Duygu, Tugba Sari, Turan Kaya, Nermin Bulut

**Affiliations:** 1 Public Health Institution of Turkey, Ankara, Turkey; 2 Tokat State Hospital, Department of Infectious Diseases and Clinical Microbiology, Tokat, Turkey; 3 Gaziosmanpasa University, Faculty of Medicine, Department of Microbiology and Clinical Microbiology, Tokat, Turkey

**Keywords:** Crimean-Congo hemorrhagic fever, Haemorrhagic fever, Brucellosis, Zoonosis

## Abstract

**Background:**

Crimean-Congo hemorrhagic fever (CCHF) is a fatal zoonotic viral disease caused by infection with a tick-borne virus of the genus *Nairovirus*. In this study, we investigated the incidence of brucellosis in patients diagnosed with CCHF.

**Methods:**

Overall, 169 patients hospitalized with an initial diagnosis of CCHF were included in 2011 in Tokat/Turkey. Immunoglobulin M (IgM) antibodies and/or PCR results were used in the laboratory diagnosis of CCHF, while plate and standard tube agglutination (STA) tests were used to diagnose brucellosis.

**Results:**

Overall, 120 patients (79%) with positive PCR tests were diagnosed with CCHF. Five (4.16%) were also diagnosed with brucellosis based on the positive plate and STA test results. Four patients (2.36%) had negative CCHF PCR and positive STA test results.

**Conclusion:**

Brucellosis and CCHF can mimic each other and that all patients with CCHF or brucellosis should be screened for both conditions.

## Introduction

Crimean-Congo hemorrhagic fever (CCHF) is a viral disease occurring in different regions worldwide and may be life threatening due to the manifestations of fever accompanied by hemorrhage. It is a zoonosis caused by *Nairoviruses* transmitted via *Hyalomma* ticks (1, 2). This disease, observed in more than 30 countries, including countries in Asia, Europe, and Africa, results in a mortality rate of 5.4–80% (3, 4). Tokat Province in northern Turkey is in an endemic area for CCHF, which is also endemic in the middle and eastern parts of the country.

CCHF is characterized by fever, malaise, sweating, anorexia, and arthralgia, and various nonspecific symptoms following an incubation period lasting less than a week. As the disease progresses, it may potentially manifest disseminated intravascular coagulation (DIC) and shock (5).

Brucellosis caused by *Brucella* bacteria represents a zoonosis transmitted to humans via the body fluids and secretions of animals such as sheep, goats, cows, buffalos, and pigs and via dairy products made with contaminated milk. After a 2–3 week incubation period, non-specific symptoms that include fatigue, lack of appetite, muscle and joint pain, and subfebrile fever may occur. Depending on the part of the body affected by the infection, different clinical characteristics may manifest (6, 7).

As the symptoms of brucellosis are non-specific, differential diagnoses are difficult and misdiagnoses are frequent. This trial designed to assess the incidence of brucellosis among patients with a preliminary diagnosis of CCHF and the clinical conditions resulting from the co-existence of the two zoonoses.

## Materials and Methods

This is a prospective cohort study implemented from Apr 2011 and Jul 2011 in Tokat, Turkey. One hundred and sixty-nine in-patients monitored at the Tokat State Hospital with a preliminary diagnosis of CCHF were enrolled in the trial. All the biochemical analyses were performed by auto-analyzer and complete blood counts were performed by automatic hemocounter at central laboratory of our hospital.

The Public Health Institution of Turkey routinely submitted case definition forms and serum samples obtained from the suspected cases (the first sample) to the National Reference Laboratory.

All patients preliminarily diagnosed with CCHF based on the case definition criteria were hospitalized and followed up, as required by the regulations. Diagnosis of CCHF and brucellosis requires satisfaction of the following criteria 1) Compatible clinical picture, 2) Diagnosis of CCHF based on detection of CCHF IgM antibodies by ELISA and/or CCHFV RNA with rreverse transcription polymerase chain reaction assays and direct sequence analyses (8, 3), 3). All patients underwent a *Brucella* Rose-Bengal slide agglutination test, with blood cultures performed for patients with a positive result based on a standard tube agglutination (STA) test and/or Coombs’ tests (titers≥1/160) (9, 10). We used a commercial kit (Cromatest, Knickerbocker Laboratories, Barcelona, Spain) for the SAT. The Coombs’ test used anti-human gamma globulin sera (Ortho Diagnostic Systems, Madrid, Spain) to detect blocking antibodies. In cases of positive sera, we performed serial tube dilutions that ranged from 1:10 to 1:1240. The BACTEC 9050 blood culture system (Becton, Dickinson and Company, USA) was used to culture Brucella. The histories and physical examination results of the patients diagnosed with brucellosis based on the clinical and laboratory findings were recorded. The age, gender, white blood cell and platelet counts, and the aspartate aminotransferase (AST) and alanine aminotransferase (ALT) levels of the patients were recorded.

Statistical analyses were conducted using SPSS ver. 17.0 for Windows (Inc., Chicago, IL, USA). Qualitative data were expressed as number (percentage) and mean comparisons for continuous variables were performed using independent-group *t*-tests.

## Results

Ninety patients (53.2%) were men, and the mean age of the patients was 32.8±9.21yr. The *Brucella* STA test was positive for nine of the 169 patients (5.02%). These nine patients were living in rural areas. They all had a history of stockbreeding and consumption of unpasteurized milk products. None had a history of brucellosis.

One hundred and twenty patients with positive CCHF PCR results were diagnosed with CCHF (79%). Five of those diagnosed with CCHF also had positive Brucella slide and tube agglutination test results (4.16%).

All patients had general symptoms of infection and fever (100%). One of the patients had sacroiliitis (11.1%) and two had splenomegaly (22.2%). Four of the 169 patients hospitalized with an initial diagnosis of CCHF was found to be CCHF PCR negative and *Brucella* STA positive (2.36%). All patients had thrombocytopenia and leukopenia. Two patients had anemia. Five patients had elevated ALT levels and eight patients had elevated AST levels. The demographic characteristics and laboratory results of the patients are presented in Table 1.

**Table 1.** Demographic characteristics and laboratory results of the patients diagnosed with brucellosis

**Patient number**	**1**	**2**	**3**	**4**	**5**	**6**	**7**	**8**	**9**
**Age (yr)**	62	23	35	46	22	42	40	46	52
**Gender**	Male	Male	Male	Male	Female	Male	Female	Female	Male
**Living area of the patients**	Rural	Rural	Rural	Rural	Rural	Rural	Rural	Rural	Rural
**Contact with ticks**	Yes	No	Yes	Yes	Yes	Yes	Yes	Yes	Yes
Milk^*^	Yes	Yes	Yes	Yes	Yes	Yes	Yes	Yes	Yes
**ALT U/L**	55	36	27	127	40	75	65	128	69
**AST U/L**	76	66	30	164	98	82	57	164	72
**WBC 1 count (10^9^/L)**	2400	2300	2700	2000	1600	3300	4300	2000	5200
**WBC 2 (10^9^/L)**	2100	1800	2000	2000	1600	3200	3500	2000	4500
**Hb**	12.2	11.6	13.9	14.9	13.8	14	11.2	14.9	13.6
**Platelets 1 (10^9^/L)**	60 000	115000	139000	59000	70000	112000	112000	59000	120000
**Platelets 2 (10^9^/L)**	42 000	61000	58000	55000	26000	90000	87000	59000	79000
**CCHF PCR**	Positive	Positive	Positive	Positive	Positive	Negative	Negative	Negative	Negative
***Brucella* STA**	1/1280	1/640	1/1280	1/320	1/640	1/320	1/1280	1/640	1/1280
**Blood Culture**	Sterile	Sterile	Sterile	Sterile	Sterile	Sterile	Sterile	Sterile	*Brucella* spp.

* Consumption of unpasteurized milk and milk products

ALT: Alanine aminotransferase, AST: Aspartate aminotransferase, WBC 1: Initial white blood cell count on referral, WBC 2: Lowest white blood cell count, Hb: Haemoglobin, Platelet 1: Initial platelet value, Platelet 2: lowest platelet value, STA: Standart tube agglutination.

Normal values: AST: 15–37 IU/L, ALT: 30–65 IU/L, WBC: 4. 800–10.800mm^3^, Hb: 12–17g/dL, Platelet: 150000–400000mm^3^

## Discussion

This trial showed that zoonotic diseases can coexist due to similar modes of transmission and that brucellosis should not be ruled out in patients with CCHF. In this trial assessing the incidence of brucellosis in patients with CCHF, five patients had both of the diseases (4.16%). The PCR results of the four patients with a preliminary diagnosis of CCHF were negative, these four patients were diagnosed with brucellosis. We believe the patients with a preliminary diagnosis of CCHF should also be investigated for brucellosis. CCHF is a zoonotic disease transmitted by *Hyalomma* ticks (1).

Brucellosis is a zoonosis transmitted via body secretions of animals with brucellosis and by the consumption of milk and dairy products not boiled or pasteurized (11). Farmers, shepherds, veterinarians, butchers, and laboratory staff are at risk for transmission (12). Both diseases are more commonly observed in people who deal with stockbreeding and live in rural areas. The most common patient complaints upon presentation with CCHF include fever, fatigue, diffuse body pain, and lack of appetite. Patients less commonly present with haemorrhage and rash compared to nausea and/or vomiting, diarrhoea, and abdominal pain (1).

Brucellosis is a disease that can involve any organ and tissue and therefore may manifest with different symptoms and findings (6, 12). Because the symptoms of brucellosis are nonspecific, differential diagnoses are difficult and misdiagnoses are frequent. Patients most commonly present with fever, fatigue, sweating, joint pain, and lack of appetite. As the disease may involve various organs, the physical examination findings can also be variable (6). The most common findings in brucellosis are fever, splenomegaly, hepatomegaly, lymphadenomegaly, and arthritis. This disease may involve all the systems and may manifest with complications. Osteoarticular involvement is most common (13). In this trial, all patients developed at least two of the general infection signs of fever, fatigue, and lack of appetite.

Leukopenia, thrombocytopenia, and anaemia may develop in CCHF (14). Similar laboratory findings can also be seen in brucellosis as a result of bone marrow suppression (13). The negative CCHF PCR results and the positive *Brucella* STA test results obtained for four of the patients admitted with a preliminary diagnosis of CCHF with symptoms of fever, general infections signs, and bicytopenia who presented due to tick contact demonstrated the importance of performing brucellosis tests in patients monitored for suspected CCHF.

CCHF is seasonal and occurs between Apr and Sep, while brucellosis may occur in any season. Therefore, we believe that seasonal features should also be considered in establishing a diagnosis and that these two diseases could co-exist during the summer. The diagnosis of CCHF is based on virus isolation in the cell culture, serologic methods (immunofluorescence assays [IFAs], ELISAs) and reverse transcription PCR (1). The most common method of diagnosing brucellosis is the standard tube agglutination (STA) test. Standard tube agglutination test is an inexpensive, convenient method with a reported sensitivity of 94%. The gold standard in diagnosis is the growth of bacteria in culture (15). All the patients were diagnosed based on PCR and/or IgM results. As the patients were hospitalized with a preliminary diagnosis of CCHF, the blood cultures could not be obtained routinely. One of the patients for whom blood culture was conducted after a brucellosis diagnosis was established exhibited *Brucella* growth in the blood culture.

Debate on the treatment of CCHF is ongoing. While some publications show ribavirin is beneficial, others indicate its lack of efficacy (16–19). Ribavirin treatment has been shown not to reduce mortality in the treatment of CCHF (20). In this trial, ribavirin was not administered to patients diagnosed with CCHF; rather, symptomatic treatment was administered. Double or triple combinations of doxycycline, streptomycin, and rifampicin are recommended to treat brucellosis (12). In this trial, all these three drugs were administered to the patients with osteoarticular involvement. Other patients were given doxycycline and rifampicin. All patients recovered. Brucellosis can show great similarity with hematologic and zoonotic diseases, such as CCHF (21–23).

## Conclusion

Brucellosis should be considered in the differential diagnosis of pancytopenia, treatment-resistant immune thrombocytopenia, and viral hemorrhagic disease, especially in countries where brucellosis is endemic.
